# Transparent Meta-Analysis: Does Aging Spare Prospective Memory with Focal vs. Non-Focal Cues?

**DOI:** 10.1371/journal.pone.0016618

**Published:** 2011-02-03

**Authors:** Bob Uttl

**Affiliations:** Department of Psychology, Mount Royal University, Calgary, Canada; Leicester University, United Kingdom

## Abstract

**Background:**

Prospective memory (ProM) is the ability to become aware of a previously-formed plan at the right time and place. For over twenty years, researchers have been debating whether prospective memory declines with aging or whether it is spared by aging and, most recently, whether aging spares prospective memory with focal vs. non-focal cues. Two recent meta-analyses examining these claims did not include all relevant studies and ignored prevalent ceiling effects, age confounds, and did not distinguish between prospective memory subdomains (e.g., ProM proper, vigilance, habitual ProM) (see Uttl, 2008, *PLoS ONE*). The present meta-analysis focuses on the following questions: Does prospective memory decline with aging? Does prospective memory with focal vs. non-focal cues decline with aging? Does the size of age-related declines with focal vs. non-focal cues vary across ProM subdomains? And are age-related declines in ProM smaller than age-related declines in retrospective memory?

**Methods and Findings:**

A meta-analysis of event-cued ProM using data visualization and modeling, robust count methods, and conventional meta-analysis techniques revealed that first, the size of age-related declines in ProM with both focal and non-focal cues are large. Second, age-related declines in ProM with focal cues are larger in ProM proper and smaller in vigilance. Third, age-related declines in ProM proper with focal cues are as large as age-related declines in recall measures of retrospective memory.

**Conclusions:**

The results are consistent with Craik's (1983) proposal that age-related declines on ProM tasks are generally large, support the distinction between ProM proper vs. vigilance, and directly contradict widespread claims that ProM, with or without focal cues, is spared by aging.

## Introduction

**Figure 1:**
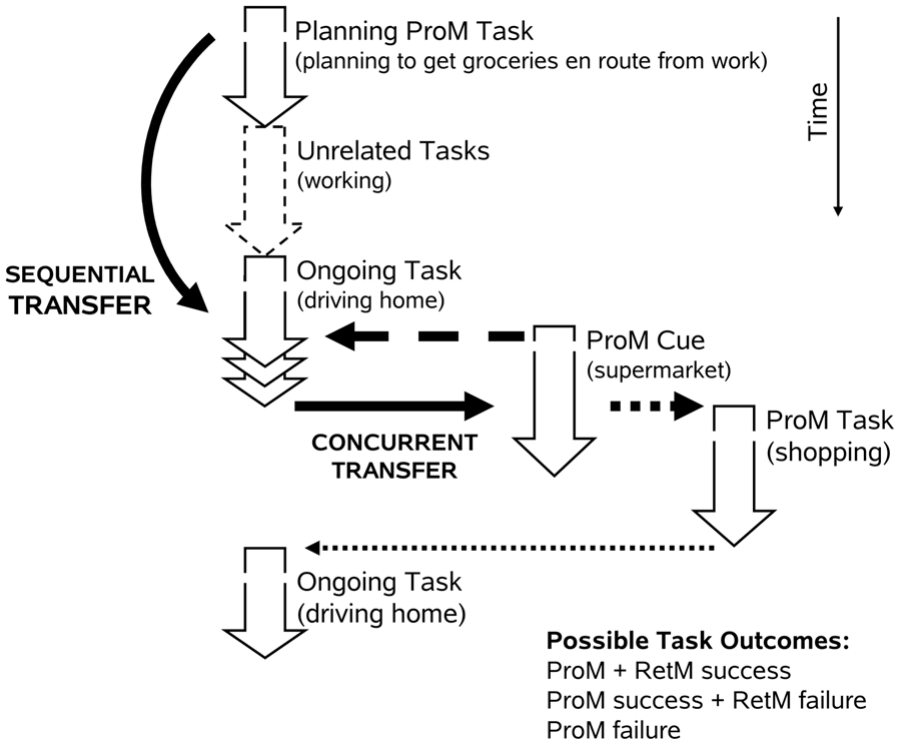
A typical situation requiring ProM proper is to buy groceries en route home form work. We make a plan to get groceries en route from work, engage in unrelated activities (work), and the function of ProM proper is to bring the plan back to consciousness at the right time and place, while driving home, in response to the ProM Cue (supermarket) (from Uttl [2]).

Prospective memory (ProM) is the ability to become aware of a previously-formed plan at the right time and place, for example, becoming aware that one wishes to mail a letter while passing by a post office, or that one wishes to buy groceries while passing by a supermarket (see Figure 1) [1]–[3]. Several important distinctions have been made in the literature on prospective memory. First, Graf and Uttl [1], [2] distinguished between different subdomains of prospective memory: prospective memory proper or episodic prospective memory (cf. Harris [4]), vigilance/monitoring, and habitual prospective memory. Prospective memory proper brings back to awareness previously-formed plans and intentions at the right place and time so that we can act upon those plans and intentions. For example, it is ProM proper that brings back to consciousness the plan to mail a letter when approaching the post office. Vigilance/monitoring differs from prospective memory proper in that the plan remains in consciousness. To illustrate, an air-traffic controller maintains a plan — to issue orders to maintain the separation of planes — in consciousness and watches out for cues to issue such orders. Although this distinction between ProM proper and vigilance/monitoring is widely recognized [1], [2], [5]–[8], it is rarely made explicit and readers must carefully read method sections to determine if a specific study concerned ProM proper vs. vigilance/monitoring [2]. In habitual prospective memory, as in prospective memory proper, a plan is made, leaves consciousness, and then must be brought back into consciousness at the right place and time. However, in contrast to ProM proper, the plan must be brought back to consciousness repeatedly whenever the ProM cue calls for the plan's performance, and in contrast to vigilance/monitoring, the plan leaves consciousness between successive occurrences of ProM cues. A classic example of habitual prospective memory use is of taking one's medication every day at bedtime. Secondly, Harris [4] and others have distinguished between event-cued and time-cued prospective memory. In event-cued prospective memory the ProM cue is an event, such as passing by the post office en route home, whereas in time-cued prospective memory the ProM cue is a time, for example to take one's medication daily at 9:00 a.m.

For over twenty years, researchers have debated whether prospective memory declines with aging or is spared by aging. Craik's [9], [10] theoretical analysis suggesting that age-related declines in prospective memory would be large – at least as large, or larger, than age-related declines in retrospective memory – was quickly opposed by Einstein and McDaniel's [11] claim that ProM is an “exciting exception to typically found age-related decrements in memory” (p. 724). Almost twenty years later, McDaniel and colleagues [12] summarized the extant research with the following: “Although the pattern of age-related effects is mixed, a significant number of studies show little or no age-related decrements in prospective memory performance on this [typical] event-based prospective memory task” (p. 823).

Most recently, in an attempt to explain this “puzzle of inconsistent age-related declines in prospective memory” [13], McDaniel and Einstein [13], [14] introduced the distinction between focal and non-focal cues, arguing that aging spares prospective memory with focal cues but impairs prospective memory with non-focal cues. For focal cues, the ongoing task requires processing of cue features relevant to the ProM plan, whereas for non-focal cues the ongoing task does not require processing of information relevant to the plan. To illustrate, encountering and talking to a friend to whom one intends to tell something is an example of a focal cue, whereas catching a glimpse of one's friend at a party while talking to someone else is an example of non-focal cue (see McDaniel, Einstein, & Rendell [13], p. 142). McDaniel, Einstein, and their colleagues argue that prospective memory with focal cues does not decline with aging because retrieval of the plan in response to the appearance of a focal cue is “automatic”, “reflexive”, and “obligatory” [13]–[15].

In a comprehensive transparent meta-analysis of previous research, Uttl [2] has recently demonstrated that, for studies conducted under controlled laboratory conditions, prospective memory performance declines with aging for event-cued prospective memory proper (*d* = −1.13), event-cued vigilance/monitoring (*d* = −0.77), and time-cued vigilance/monitoring (*d* = −0.96), whereas for studies conducted in natural settings, prospective memory task performance improves with aging for time-cued prospective memory proper (*d* =  +0.53) and time-cued habitual prospective memory (*d* =  +0.76). Thus, the cumulative findings from laboratory studies are consistent with Craik's [9], [10] theoretical proposal by demonstrating that age-related declines in ProM proper are large, at least as large as age-related declines in retrospective memory, and negate any claims that prospective memory does not decline with aging. In contrast, older adults' better performance on prospective memory tasks in uncontrolled natural settings can be explained by older adults' greater reliance on compensatory strategies, external memory aids, motivation, and other factors (see for example Maylor [16] for discussion of non-cognitive variables that can explain older vs. younger adults' superior performance on ProM tasks in natural settings).

**Figure 2:**
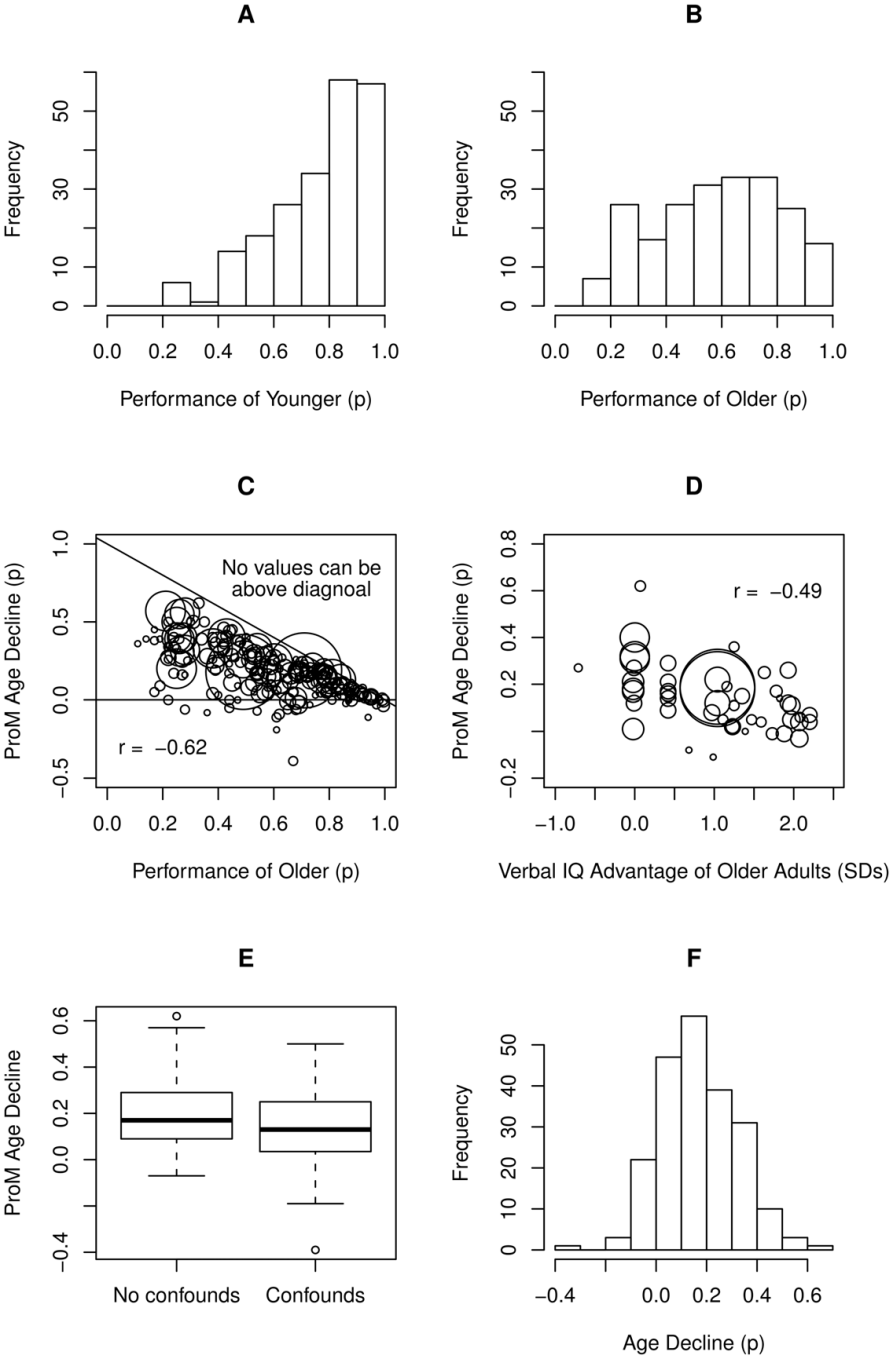
Age-related declines in event cued ProM: Summary of methodological problems and key findings. Panels A and B show that performance of younger and older adults, respectively, was perfect or nearly perfect in a substantial number of previous studies, severely restricting the size of observed age-related declines. Panel C shows the magnitude of raw ProM age-related declines as a function of the performance of older adults; it highlights that the size of the age decline is directly dependent upon the level of performance of older adults, *r* = −0.63. Panel D shows a strong relationship, *r* = −0.49, between the raw ProM age decline and one of the most common age confounds: the verbal intelligence advantage of older adults over younger adults, expressed in terms of standard deviations. Panel E shows the magnitude of raw ProM age-related declines for studies with no confounds vs. studies with age confounds favoring older adults (e.g., intelligence, ongoing task difficulty). Panel F shows the frequency distribution of raw age-related declines; it indicates that the vast majority of the previous studies have found age-related declines in event cued prospective memory assessed in laboratory (see Uttl [2]).

Equally important, Uttl's [2], [3] reviews revealed a number of methodological problems within the prospective memory research, such as: severe ceiling effects that artificially restrict the magnitude of age-related declines in individual studies; age confounds (e.g., intelligence, ongoing task difficulty) that almost always favor older adults; and failures to distinguish between the various subdomains of prospective memory. To illustrate some of the most critical methodological problems afflicting prior research on prospective memory and aging, Figure 2 focuses on event-cued prospective memory assessed under controlled laboratory conditions (collapsing across ProM proper, vigilance, and habitual ProM). Panels A and B show that the performance of younger and older adults, respectively, was perfect or nearly perfect in a substantial number of previous studies, severely limiting the size of observable age-related declines. Panel C shows the magnitude of raw ProM age-related declines as a function of performance of older adults; it highlights that the size of the age decline is directly dependent upon the performance of older adults, *r* = −0.63 (see Uttl [2], [3], [5]; see McDaniel & Einstein for an independent replication of this finding [14]). When the task was so easy that older adults scored perfect (*p* = 1.0 or 100%) no age-related declines could emerge, whereas when the task was more difficult substantial age-related declines emerged. Accordingly, one may conclude that one of the most robust findings in prospective memory literature is that the size of age-related declines depends on the researcher's ability to avoid ceiling effects [5]. Panel D shows a strong relationship, *r* = −0.49, between the raw ProM age decline and one of the most common age confounds: the verbal intelligence advantage of older adults over younger adults expressed in terms of standard deviations. Thus, one way to avoid obtaining age-related declines in prospective memory research is to compare older adults who score two standard deviations higher than younger adults on verbal intelligence measures. Panel E shows the magnitude of raw ProM age-related declines for studies with no confounds vs. studies with age confounds favoring older adults (e.g., intelligence, ongoing task difficulty). As one would expect, the data show that confounds favoring older adults reduce the size of age-related declines in ProM. Consistent with Uttl's [2], [3] reviews, Panel F shows the frequency distribution of raw age-related declines. These data highlight that, despite widespread ceiling effects and despite intelligence and ongoing task difficulty confounds favoring older adults, the vast majority of laboratory studies of event cued prospective memory assessed in laboratory conditions have found that older adults perform more poorly than younger adults.

Although unlikely in light of the overwhelming evidence of large age-related declines in event-cued prospective memory proper and event-cued vigilance/monitoring, it is still possible that there may be no age-related declines with focal cues as argued by McDaniel, Einstein, and their colleagues [13]–[15]. McDaniel and Einstein [14] recently tabulated 82 age contrasts from previously published event-cued laboratory experiments, classified each contrast as arising from the use of “focal”, “non-focal”, and “indeterminate” ProM cues, and reported that raw age-related declines were larger on non-focal than focal cues. However, they did not attempt to statistically determine whether age-related declines are actually absent with focal cues. Uttl [2] reviewed and formally analyzed McDaniel and Einstein's [14] Table 7.4 and found that age-related declines were large with both focal (*d* = −0.55, 95% CI  = −0.72, −0.36) and non-focal cues (*d* = −0.85, 95%CI  = −1.03, −0.67). More critically, the data presented by McDaniel and Einstein in Table 7.4 are biased towards minimizing age differences for the following reasons. First, McDaniel and Einstein omitted over 50% of all laboratory event-cued age contrasts reported in the literature, and they did not include all non-confounded age contrasts of event-cued prospective memory proper (all showing substantial age-related declines) (e.g., [17]–[23]), with the exception of Tombaugh et al. [24]. Given that age-related declines in ProM proper are much larger than on vigilance/monitoring [2], [3], this exclusion of ProM proper studies necessarily reduced the size of age-related declines. Second, many of the studies with focal cues listed in McDaniel and Einstein's Table 7.4 confounded age with intelligence, whereas only a few studies with non-focal cues have done so. In turn, this bias artificially reduced the size of age-related declines with focal cues. Third, McDaniel and Einstein did not consider severe ceiling effects that artificially minimize age differences [2], [3], [5]. Fourth, McDaniel and Einstein did not consider the distinction between ProM proper and vigilance/monitoring even though they themselves have endorsed this distinction on several occasions (e.g., [6]). In summary, McDaniel and Einstein's selective mini meta-analysis of ProM age-related declines with focal vs. non-focal cues has several flaws due to the biases enumerated above and discussed in detail in Uttl [2]. However, based on Uttl's [2] analysis of McDaniel and Einstein Table 7.4, we can conclude that even this biased data set selected by McDaniel and Einstein themselves strongly contradicts their claims that prospective memory with focal cues is spared by aging, and that it is not “automatic”, “reflexive”, or “obligatory”.

More recently, Kliegel, Jager, and Phillips [25] conducted a meta-analysis of event-cued prospective memory with focal vs. nonfocal cues and reported that age-related declines were larger for non-focal cues (*d* = 0.72) than focal cues (*d* = 0.54). Unfortunately, this most recent meta-analysis is also severely limited by numerous methodological problems. First, Kliegel at al. 's study omitted large number of available published age contrasts. To illustrate, Kliegel et al. excluded all “studies applying single-trial PM [ProM] tasks” (e.g., Dobbs & Rule [18]; Rendell & Thomson [17], Kliegel [19], Uttl et al. [21]), excluded all studies using continuous measures of prospective memory (e.g., Uttl [26]), and excluded all studies published in book chapters (e.g., Graf, Uttl, & Dixon [20]), with the exception of a not-yet-published study by Maylor et al. (cited in [25]) as these “authors declared that they did not intend to submit the study to a journal” [25]. Interestingly, the authors included “in preparation”, “submitted”, and “in press” works from their own labs (see Kliegel et al., Supplemental Table 1). Second, Kliegel et al. [25] did not consider methodological problems with prospective memory studies enumerated and discussed by Uttl [2], [3], [5] including widespread ceiling effects that reduce age differences and standard deviations, invalidate reliabilities and correlations, and in turn, invalidate any effect size indexes calculated from group means and standard deviation such as Hedges' *d* used by the authors. Third, Kliegel et al. [25] disregarded age confounds, including intelligence and ongoing task difficulty, and analyzed confound-free and age confounded studies mixed together. Fourth, Kliegel et al. [25] did not take into account that age-related declines vary across subdomains of ProM. Thus, Kliegel et al. 's [25] results and conclusions are an artifact of a particular blend of selected confound free and age confounded studies from various subdomains of ProM mixed together and analyzed by an effect size index that is inappropriate for ceiling-limited age contrasts.

**Table 1:** Age contrasts excluded from the meta-analysis.

1^st^ Author & Year	Exp.	Condition	n_y_	n_o_	M_y_	M_o_	Notes
Huppert '00 [55]	1	name & address*	2992	191	0.68	0.20	ps
Mantyla '97 [30]	1	signature*	500	500	0.54	0.30	ps
Cockburn '94 [56]	1	RBMT*	44	43	**0.87**	**0.81**	id
Kliegel '00 [57]	1	RBMT*	31	31	0.48	0.60	id
Martin '03 [32]	1	RBMT*	40	40	0.63	0.83	id
Bailey'10 [33]	1	Exp-controlled	73	30	**0.81**	0.60	etn
Bailey'10 [33]	1	Exp-controlled first only*	73	30	0.74	0.53	etn
Logie'09 [34]	1	smiley cue present*	8548	85	0.65	0.33	eto
Logie'09 [34]	1	smiley cue absent*	8548	85	0.57	0.19	eto
Logie'09 [34]	1	end temporal cue	8548	85	0.58	0.19	eto
Logie'09 [34]	1	later temporal cue	8548	85	0.65	0.32	eto

*Notes.* Means limited by severe ceiling effects are printed in bold; ps  =  population based study (e.g., older adults disproportionately suffering from Alzheimer's disease and/or dementia); id  =  items differ across individuals; etn  =  experimental task conducted in naturalistic settings (i.e., no control over what people were actually doing, for example, whether they used external reminders); etw  =  experimental task completed online (i.e., no control over who participated and what they were actually doing, for example whether they used external reminders);

*identifies independent age contrasts (see Methods).

Accordingly, the present meta-analysis has three aims. The first aim is to determine if event-cued prospective memory with focal cues is spared by aging as argued by McDaniel, Einstein, and their colleagues [13], [14]. The second aim is to examine whether the size of age-related declines with focal vs. non-focal cues varies with prospective memory subdomains (ProM proper vs. vigilance/monitoring). The third aim is to determine whether age-related declines with focal cues are smaller than age-related declines in retrospective memory. Finding that ProM with focal cues does not decline with age would support McDaniel and Einstein's [13],[14] claim that there are no age-related declines in ProM with focal cues as well as their theory that retrieval of prospective memory plan in response to focal cues is “automatic”, “reflexive”, and “obligatory,” whereas a finding of substantial age-related declines even with focal cues would contradict their claims and theories. Moreover, if age-related declines vary more within prospective memory subdomains and age confounds (e.g., intelligence, ongoing task difficulty) than within the focal vs. non-focal cue distinction, the results would suggest that at least some prospective memory researchers have been focusing, metaphorically, on the wrong tree or even the wrong forest in their attempts to explain, what they believe, are inconsistent age-related declines across studies.

Importantly, to minimize biases and artificial reductions in estimated effect sizes arising from methodological and measurement issues with primary data including widespread ceiling effects, low reliability, and the dichotomous nature of most of the prospective memory indexes, the present meta-analysis employs three ways of analyzing the data: graphical meta-analysis combined with effect size model fitting (see Uttl [2]), robust outcome count meta-analysis, and traditional meta-analysis using *d*_probit_ rather than the inferior *d*_p_ or *d*_phi_ based methods that derive *d* from means, standard deviations, or *t*s, *p*s, and *F*s (see [2], [27]).

## Methods

### Selection of Studies Included in Meta-Analysis

**Figure 3:**
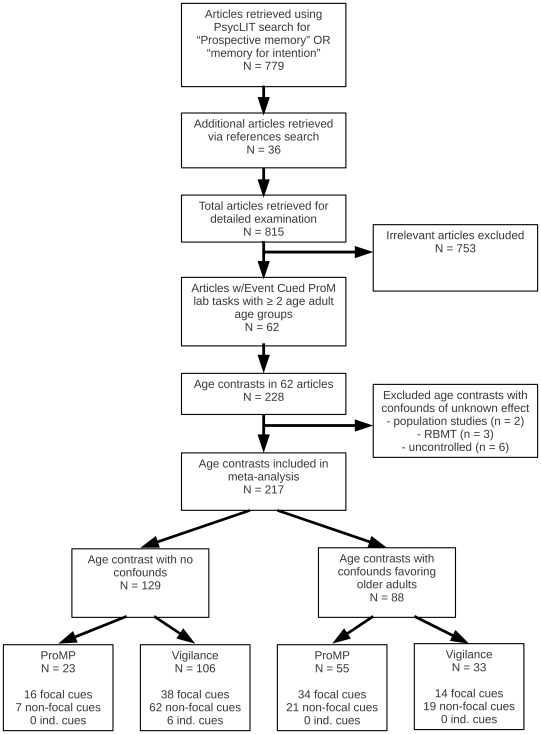
Flowchart for selection of studies and age contrasts in the present study.

Figure 3 depicts the search for relevant studies; the search proceeded in several steps, closely following the method employed by Uttl [2]. First, the PsycLIT database was searched from the earliest available date to the end of March 2010 for the following terms: “prospective memory” and “memory for intentions” and these two searches were combined with OR operator. Second, the references in Birt [28], Henry et al. [29], Uttl [2], Kliegel et al. [25] were examined for potentially relevant articles and the identified articles were examined for relevance. Next, the references in all relevant articles and book chapters, retrieved by any method were examined for potentially relevant articles and the identified articles were examined for relevance. This search yielded 815 potentially relevant articles.

The full text of all potentially relevant articles was examined for studies that reported performance on an event cued prospective memory task in laboratory settings for at least one group of younger and one group of older adults; the participant groups were healthy and without any diseases known to affect cognition (e.g., dementia); at least the mean performance for each age group was provided; and the studies were written in English. Tasks were considered to be prospective memory tasks if they required participants to perform some action in the future without any prompting from experimenters. For a few studies with more than two age groups spanning the adult lifespan, groups younger than 60 years of age were collapsed into the younger group, and groups older than 60 years of age were collapsed into the older group. This examination identified 62 articles, each reporting at least one age contrast conforming to the inclusion criteria above, and yielding 228 age contrasts in total.

Two age contrasts were excluded from the meta-analysis because age was confounded with conditions known to negatively affect cognition. For example, Mantyla and Nilsson [30] conducted a population based study of prospective memory and as a result many of their older participants scored withing the impaired range on the Mini Mental State Examination [31], a quick index of possible dementia. Three age contrasts were excluded because an examiner asked participants for their belongings and the participants' prospective memory task was to ask for their belongings at the end of the experiment (e.g., [32]). Given that the belongings turned over by participants differed across studies and participants, and likely also in terms of personal importance, it is unclear what the effect of this confound may be. Finally, six age contrasts were excluded because participants performed experimental like tasks in uncontrolled naturalistic settings (e.g., home, online) and little is known about the participants and/or how they performed the tasks (e.g., [33], [34]). To illustrate, in an ingenious study by Logie and Maylor [34], participants self-selected themselves to complete various memory tasks linked from the BBC website including a single trial ProM proper task. Thousands of people participated (73,018) and the results showed large age-related declines across the adult life span. However, the study can be criticized because, for example, we do not know much about the participants (e.g., their verbal intelligence), their state (e.g., sober, tired), and we do not know how they performed the task due to the uncontrolled setting. The excluded age contrasts, including the number and performance of younger and older adults are listed in Table 1 (all of the excluded contrasts show age-related declines in prospective memory except two). Accordingly, 217 age contrasts remained available for meta-analysis.

### Classification of Age Contrasts Included in Meta-Analysis

#### Age-Related Confounds Favoring Older Adults

The methodological review of previously published studies reveals that a large proportion of studies and age contrasts are severely limited by age confounds favoring older adults [2], [3] (see Figure 2). Thus, each age contrast was classified into one of the two categories: *age contrasts with no confounds* and *age contrasts with confounds favoring older adults* (e.g., ongoing task confounds favoring older adults, intelligence confounds favoring older adults).

Ongoing task confounds favoring older adults were introduced by Einstein and McDaniel [11] who made the ongoing task easier for older adults relative to younger adults; this design was subsequently adopted by a number of other investigators (e.g., [12], [35]–[38]). However, making the ongoing task easier for older adults artificially reduces the size of age differences and makes it impossible to disentangle the effects of aging from the effects of giving older adults an easier ongoing task (see Uttl [2] for a discussion of this issue).

Intelligence confounds favoring older adults refer to designs where highly intelligent older adults were compared to less intelligent younger adults. Since intelligence is positively correlated with prospective memory performance [39], [26], [35], this confound is also likely to artificially reduce the size of age-related declines. Indeed, as seen in Figure 2, Panel D, the intelligence advantage of older adults is moderately strongly and negatively correlated with the size of age-related declines. The affected studies include Einstein and McDaniel [11]; Cherry and LeCompte [35]; Reese and Cherry [40]; Cherry and Plauche [38]; Farrimond, Knight, and Titov [41]; Kvavilashvili et al. [37] and others. For the purposes of this article, the data are considered confounded with intelligence if older adults score more than 1.0 standard deviation above the ability of younger adults.

#### Prospective Memory Proper, Vigilance, and Habitual Prospective Memory

Consistent with the definitions above, each prospective memory task was classified as measuring prospective memory proper, vigilance/monitoring, or habitual prospective memory. Tasks that included a time delay or intervening task between prospective memory instructions and commencement of an ongoing task were classified as measuring prospective memory proper whereas tasks that included no delay between instructions and the ongoing task were classified as measuring vigilance/monitoring. This classification is consistent with the view expressed by many leading researchers in the field [1], [2], [6]–[8]. To illustrate, Marsh et al. [7] explain that “this task was merely a distractor task placed between the prospective memory instruction and the onset of the rating [ongoing] task so that the prospective task did not become vigilance task…” (p. 304). Similarly, Shapiro and Krisnan [8] note that “this delay [15 min] has been shown to be sufficient to clear short-term memory and to ensure that it is not treated as a vigilance task…” (p. 174). If a prospective memory proper task was to be executed repeatedly in response to the same cue and with the plan likely to leave consciousness between successive presentation of the cue, the task was classified as habitual prospective memory (see Uttl [2]).

#### Focal vs. Non-focal Cues

Focal cues are cues that participants must work with as part of the ongoing task, whereas non-focal cues are cues that need not be processed by participants during the course of an ongoing task. In other words, focal cues carry information relevant for performing an ongoing task, whereas non-focal cues do not provide any information relevant to performance of an ongoing task [13], [14]. By this definition, a questionnaire that a participant is required to complete is considered a focal cue if prospective memory instructions require the participant to perform some action when they are presented with the questionnaire. In contrast, the color of a toy is considered a non-focal cue when the ongoing task requires participants to sort toys into semantic categories but does not require them to attend to each toy's color. Consistent with these definitions and examples, prospective memory cues were classified as focal or non-focal for each age contrast.

The cue classification as focal vs. non-focal was compared to the cue classifications in the two previous meta-analyses by McDaniel and Einstein [14] and Kliegel et al. [25] using percentage agreement and Krippendorff's Alpha [42]. The Krippendorff's alpha measures the degree of inter-coder agreement or inter-rater reliability, with 1 indicating perfect reliability, 0 indicating the absence of reliability, and negative values indicating the systematic disagreement. The values above 0.80 are generally considered excellent. The cue classification agreement with McDaniel and Einstein' s classification of the cues was excellent: percentage agreement was 91.6% and Krippendorff's Alpha was 0.84. Similarly, the cue classification agreement with Kliegel et al. 's cue classification was also excellent: percentage agreement was 94.6% and Krippendorff's Alpha was 0.89.

### Meta-Analysis Methodology

#### Multiple Effect Sizes from Single Studies

Effect sizes were calculated for each age contrast, that is, for each reported condition with both young and older adults. However, to satisfy an independence assumption for the application of meta-analysis, each participant could contribute to only one age contrast for statistical analysis purposes. Thus, when one group of participants was tested under two different conditions, the following criteria were used to select one condition to be included in the statistical analyses: (1) condition which was administered first was preferred; (2) the condition with the smaller retrospective memory load was preferred; (3) the condition with more lenient scoring was preferred (see [1], [2], [21]); and (4) if the preceding criteria were insufficient to unambiguously choose a condition, the condition was selected at random.

#### Data Visualization and Modeling

Following Uttl [2], data visualization and modeling techniques were used to determine effect size estimates that are the least affected by ceiling effects, skewed standard deviations, and other distribution problems that are widespread in prospective memory research [2], [3]. Specifically, the performance of younger adults was plotted as a function of the performance of older adults and then the best fitting theoretical effect size curve and associated effect size was determined using double variate squared error minimization methods, both with and without weighting each point by its sample size. This modeling method minimizes the influence of ceiling-limited data as data points close to either the floor or ceiling have a small or no effect on determination of the best fitting curve. The 95% confidence interval (CI) on fitted effects were derived and the differences between the effect sizes were tested using bootstrapping methods that are robust, conservative, and require few assumptions relative to classical meta-analytic methods [43].

#### Robust Count Techniques

Robust statistical techniques — counts and sign tests — were used to determine if specific prospective memory subdomains were affected by aging.

#### Conventional Meta-Analysis

To satisfy traditionalists, conventional meta-analytic techniques were used to estimate effect sizes. However, given the dichotomous nature of primary outcome measures in all but a few studies [1]–[3], the probit was chosen as an effect size index and then transformed to its d-equivalent *d*_probit_ [27]. Theoretical and empirical research as well as examples discussed in Uttl [2] and Sanchez-Meca et al. [27] demonstrate that *d*_probit_ underestimates the true effect size much less than *phi* to *d* transformations or indices calculated using means and standard deviation such as *d*_p_ or Hedges' *d* used in previous meta-analyses [25], [28], [29]. Although these results are not reported, the data were also analyzed using odds ratios and the odds ratios yielded nearly identical effect sizes. The *I* ^2^ measure of inconsistency between studies/age contrasts in a meta-analysis is also provided; it ranges from 0 to 100% and quantifies the percentage of total variation across the studies attributed to heterogeneity rather than chance [44]. Higgins et al. [44] suggest that *I* ^2^ values of 25% indicate low inconsistency, 50% moderate inconsistency, and 75% high inconsistency among the studies.

#### Blocking

To avoid misleading and biased results, the studies were blocked by confound (e.g., age contrasts with no confounds, age contrasts with confounds favoring older adults) and analyzed separately.

## Results

The meta-analysis included 217 age contrasts from 57 articles, representing 6,765 younger (mean age  = 24.2 years) and 5,906 older (mean age  = 71.7 years) individuals, doubling or tripling the size of the meta-analyses reported by McDaniel and Einstein [14] and Kliegel et al. [25]. Thus, the present meta-analysis represents a substantial advance over the previous meta-analyses in its comprehensive coverage of previously published research.

The search yielded 129 confound-free (Tables 2, 3, 4, 5, 6, 7) and 88 age-confounded contrasts favoring older adults (Tables 8, 9, 10, 11). The contrasts listed in Tables 2, 3, 4, 5, 6, 7, 8, 9, 10, 11 are arranged by confounds (confound free, confounded age contrasts), ProM subdomain (ProM proper, vigilance), and ProM cue status (focal, non-focal, indeterminate). The tables list the number of younger and older participants, mean performance for younger and older participants, identify specific confounds in each age-contrast, and highlight prevalent ceiling effects (values >0.80 are printed in bold) (see Uttl [2], [3], [5]).

**Table 2:** Confound Free Age Contrasts: ProMP with Focal Cues.

1^st^ Author & Year	Exp.	Condition	n_y_	n_o_	M_y_	M_o_	Notes
Cuttler '07 [58]	1	questionnaire*	110	31	**0.85**	0.73	
Cuttler '07 [58]	1	plug in phone	110	31	0.49	0.22	
Dobbs '87 [18]	1	ask for pen*	138	61	**0.97**	**0.84**	
Duchek '06 [59]	1	knowledge*	20	33	**0.87**	0.69	
Kliegel '00 [57]	1	six elements*	31	31	0.64	0.36	
Kliegel '04 [19]	1	six elements*	19	21	**0.95**	0.33	
Kliegel'08 [60]	1	initiation*	79	79	0.71	0.38	
Rendell '99 [17]	3	note-finish*	175	80	0.78	0.21	
Salthouse '04 [22]	1	red pencil*	255	75	**0.83**	0.43	
Skladzien'10 [61]	1	first*	31	31	**0.95**	0.77	
Tombaugh '95 [24]	1	6 tasks*	31	33	**0.87**	0.60	
Uttl '01 [21]	1	name*	31	23	**0.84**	0.43	
Uttl '01 [21]	1	letter	31	23	**0.94**	0.70	
Uttl '01 [21]	1	check	31	23	**0.97**	0.78	
West '88 [23]	2	message*	26	26	**0.85**	0.50	
West '88 [23]	2	check & ask	26	26	**0.81**	0.31	

*Notes.* Means limited by severe ceiling effects are printed in bold;

*identifies independent age contrasts (see Methods).

**Table 3:** Confound Free Age Contrasts: ProMP with Non-focal Cues.

1^st^ Author & Year	Exp.	Condition	n_y_	n_o_	M_y_	M_o_	Notes
Graf '02 [20]	1	visual*	60	51	8.53	10.19	cm
Kliegel'08 [60]	1	event*	79	79	0.71	0.38	
Skladzien'10 [61]	2	first naïve*	30	30	**0.92**	0.78	
Skladzien'10 [61]	2	first preexposed	30	30	**0.90**	**0.85**	
Uttl'06 [26]	1	visual*	29	18	9.97	15.53	cm
Uttl'06 [26]	1	auditory	29	18	7.06	15.05	cm
Zimmerman'05 [62]	1	identity*	80	40	**0.98**	0.78	

*Notes.* Means limited by severe ceiling effects are printed in bold; cm  =  continuous measures of ProM (higher scores indicate poorer ProM performance);

* identifies independent age contrasts (see Methods).

**Table 4:** Confound Free Age Contrasts: Vigilance with Focal Cues.

1^st^ Author & Year	Exp.	Condition	n_y_	n_o_	M_y_	M_o_	Notes
Cohen '01 [63]	1	very related	24	24	**0.92**	0.78	
Cohen '01 [63]	1	somewhat related*	24	24	**0.87**	0.72	
Cohen '01 [63]	1	unrelated	24	24	0.73	0.52	
Cohen '01 [63]	2	picture+word related	24	24	**0.96**	**0.85**	
Cohen '01 [63]	2	picture+word unrelated	24	24	**0.91**	**0.74**	
Cohen '01 [63]	2	word only related*	24	24	0.74	0.45	
Cohen '01 [63]	2	word only unrelated	24	24	0.73	0.34	
d'Ydewalle '99 [64]	1	q&a	30	30	**0.81**	0.42	
Einstein '95 [65]	2	specific cue*	11	12	**0.85**	**0.83**	
Einstein '97 [66]	1	standard*	16	16	0.71	0.53	
Einstein '97 [66]	1	demanding*	16	16	0.58	0.25	
Einstein '97 [66]	2	enc std/ret std*	16	16	0.66	0.58	
Einstein '97 [66]	2	enc std/ret dem*	16	16	0.64	0.38	
Einstein '97 [66]	2	enc dem/ret std*	16	16	0.47	0.54	
Einstein '97 [66]	2	enc dem/ret dem*	16	16	0.55	0.17	
Einstein '98 [67]	1	std att/no cue*	15	15	**0.91**	0.69	
Einstein '98 [67]	1	std att/cue*	15	15	**0.89**	0.73	
Einstein '98 [67]	1	div att/no cue*	15	15	**0.82**	0.62	
Einstein '98 [67]	1	div att/cue*	15	15	**0.81**	0.52	
Logie '04 [68]	1	low arithmetic	10	10	**1.00**	**0.98**	
Logie '04 [68]	1	high arithmetic*	10	10	**0.96**	**0.80**	
Martin '03 [32]	1	word rating*	40	40	**0.95**	0.79	
Maylor '02 [69]	1	movie*	15	15	**1.00**	**0.92**	
Maylor '02 [69]	2	related	10	10	**1.00**	**0.88**	
Maylor '02 [69]	2	unrelated*	10	10	**0.96**	**0.90**	
McDaniel'07 [13]	1	focal*	14	14	**0.89**	**0.83**	
McDaniel'07 [13]	2	focal/trial 1*	24	24	**0.92**	**0.83**	
McDaniel'07 [13]	2	focal/trial 2	24	24	**0.96**	**1.00**	
McDaniel'07 [13]	2	focal/trial 3	24	24	**0.96**	**0.92**	
McDaniel'07 [13]	2	focal/trial 4	24	24	**0.88**	**0.88**	
Rendell'07 [15]	1	focal*	72	60	**0.91**	0.77	
Salthouse '04 [22]	1	concepts	255	75	0.75	0.49	
Salthouse '04 [22]	1	pictures	255	75	**0.95**	**0.81**	
Vogels '02 [70]	1	pictures	16	14	**0.84**	0.69	
West '01b [71]	1	percep/immed	20	20	**0.86**	0.60	
West '01b [71]	1	percept/post	20	20	0.58	0.24	
West '01b [71]	1	sem/immed*	20	20	0.58	0.39	
West '01b [71]	1	sem/post	20	20	0.64	0.24	

*Note.* Means limited by severe ceiling effects are printed in bold;

*identifies independent age contrasts (see Methods).

**Table 5:** Confound Free Age Contrasts: Vigilance with Non-Focal Cues.

1^st^ Author & Year	Exp.	Condition	n_y_	n_o_	M_y_	M_o_	Notes
D'Ydewalle '01 [72]	1	low complexity*	12	12	0.62	0.17	
D'Ydewalle '01 [72]	1	high complexity*	12	12	0.70	0.73	
D'Ydewalle '99 [64]	1	faces*	30	30	**0.92**	0.73	
Einstein '95 [65]	2	general cue*	12	12	0.56	0.47	
Kidder '97 [73]	1	WM 2/ProM 1*	15	15	**0.98**	**0.98**	
Kidder '97 [73]	1	WM 3/ProM 1*	15	15	**0.82**	0.69	
Kidder '97 [73]	1	WM 2/ProM 3*	15	15	**0.97**	0.85	
Kidder '97 [73]	1	WM 3/ProM 3*	15	15	**0.90**	0.63	
Kliegel'06 [74]	1	low WM load/cue 1*	27	31	**0.89**	0.72	
Kliegel'06 [74]	1	low WM load/cue 2	27	31	**0.96**	**0.80**	
Kliegel'06 [74]	1	low WM load/cue 3	27	31	**0.89**	**0.80**	
Kliegel'06 [74]	1	low WM load/cue 4	27	31	**0.93**	**0.84**	
Kliegel'06 [74]	1	high WM load/cue1*	27	31	**0.85**	0.56	
Kliegel'06 [74]	1	high WM load/cue 2	27	31	0.74	0.60	
Kliegel'06 [74]	1	high WM load/cue 3	27	31	**0.85**	0.64	
Kliegel'06 [74]	1	high WM load/cue 4	27	31	**0.85**	0.68	
Mantyla '93 [75]	1	typical/primed	16	16	**0.86**	0.75	
Mantyla '93 [75]	1	typical/nonprime*	16	16	**0.80**	0.49	
Mantyla '93 [75]	1	atypical/primed	16	16	**0.80**	0.30	
Mantyla '93 [75]	1	atypical/nonprime	16	16	0.48	0.22	
Mantyla '94 [76]	1	typical*	18	18	0.79	0.65	
Mantyla '94 [76]	1	atypical	18	18	0.65	0.26	
Maylor '93 [77]	1	block 1*	43	43	0.69	0.68	
Maylor '93 [77]	1	block 2	43	43	**0.83**	0.66	
Maylor '93 [77]	1	block 3	43	43	**0.87**	0.69	
Maylor '93 [77]	1	block 4	43	43	**0.92**	0.71	
Maylor '96 [39]	1	block 1*	56	59	0.57	0.26	
Maylor '96 [39]	1	block 2	56	59	0.65	0.25	
Maylor '96 [39]	1	block 3	56	59	0.67	0.27	
Maylor '96 [39]	1	block 4	56	59	0.60	0.28	
Maylor '98 [78]	1	block 1*	45	59	0.65	0.26	
Maylor '98 [78]	1	block 2	45	59	0.75	0.25	
Maylor '98 [78]	1	block 3	45	59	**0.81**	0.26	
Maylor '98 [78]	1	block 4	45	59	**0.84**	0.28	

*Note.* Means limited by severe ceiling effects are printed in bold;

*identifies independent age contrasts (see Methods).

**Table 6:** Confound Free Age Contrasts: Vigilance with Non-Focal Cues (Continued).

1^st^ Author & Year	Exp.	Condition	n_y_	n_o_	M_y_	M_o_	Notes
McDaniel'07 [13]	1	nonfocal*	14	14	0.43	0.40	
McDaniel'07 [13]	2	nonfocal/trial 1*	24	24	0.71	0.63	
McDaniel'07 [13]	2	nonfocal/trial 2	24	24	0.71	0.54	
McDaniel'07 [13]	2	nonfocal/trial 3	24	24	0.63	0.58	
McDaniel'07 [13]	2	nonfocal/trial 4	24	24	0.42	0.38	
Park '97 [79]	1	6-event*	16	16	**0.94**	0.71	
Park '97 [79]	1	12-event*	16	16	**0.92**	**0.87**	
Rendell'07 [15]	1	nonfocal*	72	60	**0.87**	0.54	
Rendell'07 [15]	2	standard*	20	20	0.76	0.39	
Rendell'07 [15]	2	simple*	20	20	0.72	0.67	
Rendell'07 [15]	2	slow*	20	20	0.62	0.68	
Salthouse '04 [22]	1	WML3*	255	75	**0.84**	0.60	
Vogels '02 [70]	1	block 1*	16	16	**0.81**	**0.81**	
Vogels '02 [70]	1	block 2	16	16	**0.91**	**0.88**	
Vogels '02 [70]	1	block 3	16	16	**0.94**	**0.97**	
Vogels '02 [70]	1	word comp	16	11	**0.94**	0.68	
Vogels '02 [70]	1	no feedback	15	13	**0.84**	**0.86**	
Vogels '02 [70]	1	feedback	15	13	**0.88**	**0.87**	
West '01a [80]	1	w/classification*	16	16	**0.95**	**0.83**	
West '01b [71]	1	sem/immed	20	20	0.41	0.24	
West '01b [71]	1	sem/post*	20	20	0.28	0.19	
West '01b [71]	1	percep/immed	20	20	**0.89**	0.58	
West '01b [71]	1	percept/post	20	20	0.78	0.38	
West '03 [81]	1	w/classification*	16	16	0.73	0.46	
West'05 [82]	1	1-back*	18	18	0.69	0.59	
West'05 [82]	1	2-back	18	18	0.59	0.65	
West '99a [83]	2	w/classification*	12	12	**0.91**	0.75	
Zollig'07 [84]	1	event*	14	14	**0.92**	0.76	

*Note.* Means limited by severe ceiling effects are printed in bold;

*identifies independent age contrasts (see Methods).

**Table 7:** Confound Free Age Contrasts: Vigilance with Indeterminate Clues.

1^st^ Author & Year	Exp.	Condition	n_y_	n_o_	M_y_	M_o_	Notes
Cohen '03 [85]	1	none displaced*	30	30	**0.82**	0.53	
Cohen '03 [85]	1	target displaced	30	30	0.71	0.54	
Cohen '03 [85]	1	cue displaced	30	30	**0.80**	0.59	
Cohen '03 [85]	2	none displaced	31	34	0.56	0.45	
Cohen '03 [85]	2	target displaced	31	34	0.61	0.39	
Cohen '03 [85]	2	cue displaced*	31	34	0.71	0.55	

*Note.* Means limited by severe ceiling effects are printed in bold;

*identifies independent age contrasts (see Methods).

**Table 8:** Confounded Age Contrasts Favoring Older Adults: ProMP with Focal Cues.

1^st^ Author & Year	Exp.	Condition	n_y_	n_o_	M_y_	M_o_	Notes
Cherry '01 [36]	1	specific cue*	16	16	**0.90**	0.50	ic,otc
Cherry '01 [36]	2	specific cue*	20	20	0.53	0.62	ic,otc
Cherry '01 [36]	3	specific/typical*	12	12	**0.86**	0.61	ic,otc
Cherry '01 [36]	3	specific/atypical*	12	12	0.78	0.75	ic,otc
Cherry '03 [38]	1	low complexity/low support/trial 1*	18	18	0.28	0.67	ic,otc
Cherry '03 [38]	1	low complexity/low support/trial 2	18	18	0.61	0.44	ic,otc
Cherry '03 [38]	1	low complexity/low support/trial 3	18	18	0.72	0.67	ic,otc
Cherry '03 [38]	1	low complexity/high support/trial 1*	18	18	0.39	0.44	ic,otc
Cherry '03 [38]	1	low complexity/high support/trial 2	18	18	0.50	0.61	ic,otc
Cherry '03 [38]	1	low complexity/high support/trial 3	18	18	0.67	0.61	ic,otc
Cherry '03 [38]	1	high complexity/low support/trial 1*	18	18	0.22	0.22	ic,otc
Cherry '03 [38]	1	high complexity/low support/trial 2	18	18	0.22	0.28	ic,otc
Cherry '03 [38]	1	high complexity/low support/trial 3	18	18	0.22	0.17	ic,otc
Cherry '03 [38]	1	high complexity/high support/trial 1*	18	18	0.44	0.44	ic,otc
Cherry '03 [38]	1	high complexity/high support/trial 2	18	18	0.56	0.22	ic,otc
Cherry '03 [38]	1	high complexity/high support/trial 3	18	18	0.72	0.22	ic,otc
Cherry '99 [35]	1	low IQ*	24	24	0.65	0.40	ic,otc
Cherry '99 [35]	1	high IQ*	24	24	0.68	0.69	ic,otc
Einstein '90 [11]	1	no aid*	12	12	0.47	0.47	ic,otc
Einstein '90 [11]	1	aid*	12	12	**0.83**	0.69	ic,otc
Einstein '90 [11]	2	familiar*	12	12	0.28	0.36	otc
Einstein '90 [11]	2	unfamiliar*	12	12	**0.83**	**0.94**	otc
Einstein '92 [86]	1	1 trg/short*	12	12	0.58	0.53	otc
Einstein '92 [86]	1	1 trg/long*	12	12	0.42	0.61	otc
Einstein '92 [86]	1	4 trg/short*	12	12	0.58	0.19	otc
Einstein '92 [86]	1	4 trg/long*	12	12	0.47	0.11	otc
Einstein '92 [86]	2	4 trg*	12	12	0.53	0.14	otc
Einstein '95 [65]	3	q&a*	18	13	**0.93**	**0.86**	otc
Kidder '97 [73]	1	DOW/ProM	90	80	0.45	0.25	exl
Kvavilashvili '09 [37]	1	red pen*	72	151	**0.90**	0.71	ic
McDaniel '03 [12]	2b	full*	12	12	**0.97**	**0.93**	otc
McDaniel '03 [12]	2b	divided	12	12	**0.87**	**0.87**	otc
Reese '02 [40]	1	low IQ*	32	32	0.59	0.51	ic,otc
Reese '02 [40]	1	high IQ*	32	32	0.64	0.65	ic,otc

*Note.* Means limited by severe ceiling effects are printed in bold; ic  =  intelligence confound favoring older adults; otc  =  ongoing task confound favoring older adults;

*identifies independent age contrasts (see Methods).

**Table 9:** Confounded Age Contrasts Favoring Older Adults: ProMP with Non-focal Cues.

1^st^ Author & Year	Exp.	Condition	n_y_	n_o_	M_y_	M_o_	Notes
Cherry '01 [36]	1	general cue*	16	16	0.54	0.29	ic,otc
Cherry '01 [36]	2	general cue*	20	20	0.43	0.27	ic,otc
Cherry '01 [36]	3	general/typical*	12	12	0.67	0.44	ic,otc
Cherry '01 [36]	3	general/atypical*	12	12	0.44	0.28	ic,otc
Jager '08 [87]	1	event*	30	32	**0.97**	**0.95**	ic
Jager'08 [87]	1	event*	30	27	**0.97**	**0.95**	ic
Kvavilashvili '09 [37]	1	color	72	151	0.67	0.49	ic
Kvavilashvili '09 [37]	1	activity	24	50	**0.96**	0.74	ic,otc
Kvavilashvili '09 [37]	1	event	24	50	0.74	0.62	ic,otc
McDaniel '03 [12]	1	5 s/unfilled*	20	20	**0.90**	0.45	otc
McDaniel '03 [12]	1	5 s/filled	20	20	**0.85**	0.35	otc
McDaniel '03 [12]	1	15 s/unfilled	20	20	**0.85**	0.48	otc
McDaniel '03 [12]	1	15 s/filled	20	20	**0.82**	0.52	otc
McDaniel '03 [12]	1	5 s/unfilled/rehearsal*	20	20	**0.90**	0.74	otc
McDaniel '03 [12]	1	5 s/filled/rehearsal	20	20	**0.85**	0.47	otc
McDaniel '03 [12]	1	15 s/unfilled/rehearsal	20	20	**0.85**	0.60	otc
McDaniel '03 [12]	1	15 s/filled/rehearsal	20	20	**0.82**	0.57	otc
McDaniel '03 [12]	2a	break/full*	40	40	**0.93**	0.79	otc
McDaniel '03 [12]	2a	break/divided	40	40	0.78	0.52	otc
McDaniel '03 [12]	2a	trivia/full	40	40	**0.82**	0.53	otc
McDaniel '03 [12]	2a	trivia/divided	40	40	0.77	0.40	otc

*Note.* Means limited by severe ceiling effects are printed in bold; ic  =  intelligence confound favoring older adults; otc  =  ongoing task confound favoring older adults;

*identifies independent age contrasts (see Methods).

**Table 10:** Confounded Age Contrasts Favoring Older Adults: Vigilance with Focal Cues.

1^st^ Author & Year	Exp.	Condition	n_y_	n_o_	M_y_	M_o_	Notes
Einstein '00 [11]	1	no del/standard	20	20	**0.97**	**0.95**	otc
Einstein '00 [11]	1	no del/divided*	20	20	**0.96**	**0.88**	otc
Farrimond'06 [41]	1	3 study trials*	20	20	**0.92**	**0.87**	ic
Farrimond'06 [41]	1	1 study trial*	20	20	**0.84**	0.79	ic
Farrimond'06 [41]	2	familiar	30	30	**0.95**	**0.91**	ic
Farrimond'06 [41]	2	unfamiliar*	30	30	**0.95**	**0.88**	ic
Farrimond'06 [41]	3	distraction*	20	20	**0.96**	**0.92**	ic
Farrimond'06 [41]	3	interruption*	20	20	**0.96**	0.77	ic
Farrimond'06 [41]	3	control*	20	20	**0.95**	**0.89**	ic
McDermott '04 [88]	1	movie*	30	30	0.73	0.58	ic
Rendell '00 [17]	1	irregular tasks*	20	20	0.78	0.42	ic
Rendell '00 [17]	1	regular tasks	20	20	**0.93**	**0.82**	ic
West '01b [71]	2	percep	12	12	**0.88**	0.67	ic
West '01b [71]	2	seman	12	12	**0.92**	0.73	ic

*Note.* Means limited by severe ceiling effects are printed in bold ; ic  =  intelligence confound favoring older adults; otc  =  ongoing task confound favoring older adults;

*identifies independent age contrasts (see Methods).

**Table 11:** Confounded Age Contrasts Favoring Older Adults: Vigilance with Non-focal Cues.

1^st^ Author & Year	Exp.	Condition	n_y_	n_o_	M_y_	M_o_	Notes
Bastin '02 [89]	1	12-event/recall absent	24	24	**1.00**	**0.99**	otc
Bastin '02 [89]	1	12-event/recall present*	24	24	**0.95**	**0.93**	otc
Bastin '02 [89]	1	6-event/recall absent	24	24	**0.99**	**1.00**	otc
Bastin '02 [89]	1	6-event/recall present*	24	24	**0.84**	0.79	otc
Einstein '00 [11]	1	delay exe/standard	20	20	**0.82**	0.77	otc
Einstein '00 [11]	1	delay exe/divided*	20	20	0.72	0.48	otc
Einstein '00 [11]	2	10s/unfilled*	24	24	**0.88**	0.42	otc
Einstein '00 [11]	2	10s/filled	24	24	0.75	0.42	otc
Einstein '00 [11]	2	30s/unfilled	24	24	**0.88**	0.44	otc
Einstein '00 [11]	2	30s/filled	24	24	0.79	0.55	otc
Knight'08 [90]	1	low distraction*	32	32	0.78	0.66	ic
Knight'08 [90]	1	high distraction	32	32	0.53	0.27	ic
Marsh'07 [91]	1	nonrepeated/first half*	35	35	0.59	0.55	ic
Marsh'07 [91]	1	nonrepeated/second half	35	35	0.66	0.69	ic
Marsh'07 [91]	2	nonrepeated/first half*	35	35	0.62	0.57	ic
Marsh'07 [91]	2	nonrepeated/second half	35	35	0.64	0.53	ic
West '01b [71]	2	seman*	12	12	0.70	0.41	ic
West '01b [71]	2	percept*	12	12	**0.92**	0.70	ic
West '99a [83]	1	w/classification*	24	24	**0.96**	0.79	ic

*Note.* Means limited by severe ceiling effects are printed in bold; ic  =  intelligence confound favoring older adults; otc  =  ongoing task confound favoring older adults;

*identifies independent age contrasts (see Methods).

**Figure 4:**
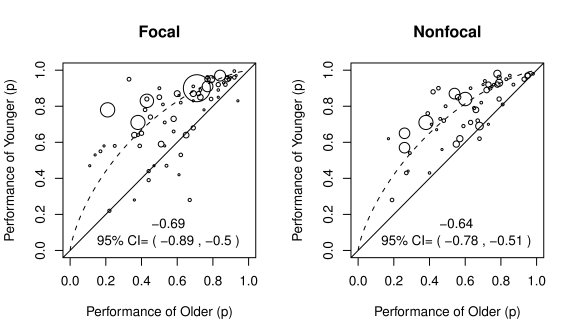
Age-related declines in ProM with focal vs. non-focal cues, disregarding ceiling effects, confounds, and subdomains. These figures include the best fitting estimated *d* derived by double variate square error minimization methods and associated 95% confidence intervals derived by bootstrapping methods. This figure highlights that the vast majority of studies in both focal and non-focal conditions show substantial age-related declines and that age-related declines with focal cues were comparable to age-related declines with non-focal cues. However, these results reflect a specific blend of ceiling-limited and age-confounded studies of ProM proper, vigilance/monitoring, and habitual ProM all mixed together despite known differences among them.

For *illustrative purposes only* and to allow comparison with the “mix everything together” approach adopted in the meta-analysis by Kliegel et al. [25], Figure 4 shows the performance of younger adults as a function of the performance of older adults with focal vs. nonfocal cues for all available contrasts, that is, *disregarding age confounds* (*e.g.*, *intelligence*, *ongoing task difficulty*) *and prospective memory subdomains* (*e.g.*, *prospective memory proper*, *vigilance*). Figure 4 includes the best fitting estimated *d* derived by double variate square error minimization methods and associated 95% confidence intervals derived by bootstrapping methods using 10,000 samples [43] and also highlights that the vast majority of studies in both focal and nonfocal conditions show substantial age-related declines and that age-related declines on focal cues [*d* = −0.69; 95% CI  = (−0.89, −0.50)] were comparable to age-related declines on non-focal cues [*d* = −0.64; 95% CI  = (−0.78, −0.51)]. However, these results reflect a particular blend of confounds and prospective memory subdomains.

**Figure 5:**
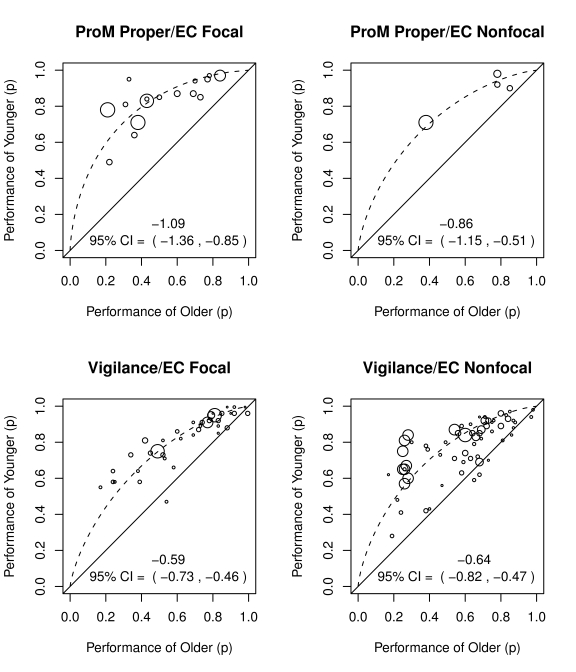
Age-related declines in ProM with focal vs. non-focal cues, for confound-free age-contrasts only. Large age-related declines are readily apparent in all of the conditions where sufficient data are available: ProM proper with focal cues, vigilance/monitoring with focal cues, and vigilance/monitoring with nonfocal cues. Moreover, for focal cues, age-related declines are much larger on ProM proper than on vigilance/monitoring, *d* difference  = 0.40 with bootstrap 95% CI  = (0.14, 0.68), and for vigilance/monitoring, age-related declines are only numerically larger with non-focal cues than with focal cues, *d* difference  = 0.05 with bootstrap 95% CI  = (−0.17,0.27).

Figure 5 shows the performance of younger adults as a function of the performance of older adults for conditions with focal vs. non-focal cues, for prospective memory proper and for vigilance (no studies of event cued habitual prospective memory were identified in the review, see Uttl [2], for a more extensive discussion of this point), *for confound free age-contrasts only*. Several findings are readily apparent from the data. First, the majority of previous confound-free age contrasts examined vigilance/monitoring and a comparatively small number of age contrasts examined prospective memory proper. Only four ProM proper age contrasts with non-focal cues and binary outcome measures were identified by the review. An additional three contrasts not shown in the figure involved continuous measures of ProM and all showed an age decline (see [26], [20]). Second, large age-related declines are readily apparent in all of the conditions where sufficient data are available: ProM proper with focal cues [*d* = −1.09; 95% CI  =  (−1.36, −0.85)], vigilance/monitoring with focal cues [*d* = −0.59, 95% CI  =  (−0.73, −0.46)], and vigilance/monitoring with non-focal cues [*d* = −0.64; 95% CI  = (−0.82, −0.47)]. (The age-related declines on the only four contrasts available for ProM proper with non-focal cues was also significant [*d* = −0.86, 95% CI  =  (−1.15, −0.51)].) Third, age-related declines show large differences between prospective memory subdomains. For focal cues, age-related declines were much larger in ProM proper than in vigilance/monitoring, *d* difference  = 0.40 with bootstrap 95% CI  = (0.14, 0.68). Fourth, age-related declines were numerically larger with non-focal vs. focal cues but the difference was not statistically significant, *d* difference  = 0.05 with bootstrap 95% CI  =  (−0.17, 0.27).

For each subdomain and type of cue (focal vs. nonfocal), Table 12 shows the number of age contrasts available (*k*) as well as a summary of the outcomes — number of age contrasts showing decline, age parity (i.e., no differences), and age improvement, for all outcomes (i.e., a participant may have contributed data to more than one condition/age contrast) and for independent outcomes only (i.e., each participant contributed data to only one condition/age contrast). In addition, for independent outcomes only, Table 12 shows the result of the robust sign test meta-analyses and the conventional random effect meta-analyses using *d*_probit_ as the effect size index, including the inconsistency index.

**Table 12:** Summary of meta-analysis for all outcomes and for independent outcomes.

	*k*	De-Eq-Im	*d*_probit_	*k* *	*N* *	De-Eq-Im*	*p* *	*d*_probit_ *	*I^2^*
**CONFOUND FREE**									
**ProMP**	23	23-0-0	-0.93	17	1,966	17-0-0	<.001	−0.92 (−1.13, −0.72)	53.1
Focal	16	16-0-0	-1.02	12	1,470	12-0-0	<.001	−1.01 (−1.24, −0.78)	44.8
Nonfocal	7	7-0-0	-0.78	5	496	5-0-0	Insuf.	Insufficient data	–
**Vigilance**	106	96-3-7	-0.68	54	2,641	49-2-3	<.001	−0.61 (−0.71, −0.49)	0
Focal	38	35-1-2	-0.66	21	829	20-0-1	<.001	−0.58 (−0.78, −0.38)	0
Nonfocal	62	55-2-5	-0.68	31	1687	27-2-2	<.001	−0.61 (−0.75, −048)	0
Indeterminate	6	6-0-0	-0.52	2	125	2-0-0	Insuf.	Insufficient data	–
**CONFOUNDED**									
**ProMP**	55	41-4-10	-0.47	33	1,381	22-3-8	.016	−0.32 (−0.51, −0.14)	25.1
Focal	34	20-4-10	-0.27	24	982	13-3-8	.383	−0.22 (−0.45,+0.01)	33.3
Nonfocal	21	21-0-0	-0.72	9	399	9-0-0	.004	−0.62 (−0.93, −0.31)	0
**Vigilance**	33	31-0-2	-0.52	19	884	19-0-0	<.001	−0.47 (−0.67, −0.27)	0
Focal	14	14-0-0	-0.52	9	400	9-0-0	.004	−0.52 (−0.84, −0.19)	0
Nonfocal	19	17-0-2	-0.48	10	484	10-0-0	.002	−0.46 (−0.72, −0.19)	8.2

*Note.* De  =  decline, Eq  =  equal, Im  =  improvement; *k*  =  number of age contrasts; *N*  =  total number of individuals; *p*  =  binomial test *p;* Insuf.  =  Insufficient data  =  fewer than 6 age contrasts ;

*Independent outcomes; *I^2^* =  inconsistency index.

The data in Table 12 are consistent with the modeling results. Looking at the independent outcomes only, both the binomial tests and *d*_probit_ show statistically significant large age-related declines in all three subdomains/focal/non-focal conditions with sufficient data: ProMP with focal cues (12 declines, 0 ties, 0 improvements; *d*_probit_ = −1.01), vigilance with focal cues (20 declines, 0 ties, 1 improvement; *d*_probit_  = −0.58), and vigilance with non-focal cues (27 declines, 2 ties, 2 improvements; *d*_probit_  = −0.61).

Finally, Table 12 shows the outcomes of studies with age confounds favoring older adults (ongoing task confounds, intelligence confounds, or both). Even though the age confounds favored older adults, the results of these confounded studies also show age-related declines in all conditions except in ProM proper with focal cues, *d*_probit_  = −0.22, 95% CI  =  (−0.45,+0.01). Not surprisingly, however, age-related declines were smaller in these age-confounded studies favoring older adults than in the studies without age confounds (see Uttl [2]).

## Discussion

The current comprehensive meta-analysis of age-related declines in ProM with focal vs. non-focal cues yielded several critical findings. First, age-related declines in ProM with both focal and non-focal cues are large. Second, age-related declines in ProM with focal cues vary across subdomains; they are large in ProM proper and smaller in vigilance. Third, age-related declines in ProM proper with focal cues (*d* = −1.09) are as large or larger than age-related declines in recall measures of retrospective memory as reported in several independent meta-analyses of retrospective memory declines (*d* = 1.01, Spencer & Raz [45]; *d* = 0.97, LaVoie & Light [46]; *d* = 0.99, Verhaeghen Marcoen, & Gossens [47]).

The substantial age-related declines in ProM with both focal and non-focal cues directly contradicts Einstein, McDaniel, and their colleagues' claims that aging spares prospective memory with focal cues, consistent with previous findings by Uttl [2] and Kliegel et al. [25]. As discussed by Uttl [2], [3], the evidence offered by McDaniel, Einstein, and their colleagues in support of their claim that prospective memory is an “exciting exception to age-related declines in memory” has been based on null findings due to (1) methodological artifacts such as ceiling effects [5], (2) intelligence confounds favoring older adults (see Figure 2), (3) ongoing task difficulty confounds favoring older adults (e.g., [11]), (4) studies with astonishingly low statistical power to detect even very large age differences in ProM (see Uttl [2] for discussion); and (5) studies of vigilance as opposed to ProM proper where age-related declines are smaller [2], [3]. Perhaps not surprisingly, claims of no age-related declines in ProM with focal cues are similarly based on data compromised by ceiling effects, intelligence age-confounds, ongoing task-age confounds, low statistical power, and studies of vigilance. When age-confounded studies are removed from the analyses and the studies are blocked by ProM subdomain, the accumulated evidence shows that age-related declines in ProM with focal cues are large in ProM proper (*d* = −1.09) and smaller but still substantial in vigilance (*d* = −0.59). In turn, the findings strongly contradict McDaniel and Einstein's claims that ProM with focal cues is spared by aging due to “automatic”, “obligatory”, or “reflexive” retrieval of the previously formed plan. On the contrary, smaller age-related declines in studies with vs. without ongoing task age confounds favoring older adults (e.g., easier ongoing tasks for older adults) suggest that retrieval of the plan requires cognitive resources and is anything but automatic, obligatory, or reflexive.

The current meta-analysis revealed much larger age-related declines in ProM with focal cues than the previous meta-analyses. As noted in the introduction, Uttl [2] reanalyzed data presented by McDaniel and Einstein [14] in their Table 7.4 and demonstrated that even McDaniel and Einstein's own very limited selection of studies and classification of ProM cues as focal vs. non-focal revealed large age-related declines in ProM with both focal and non-focal cues, contrary to their claims. However, Uttl also noted that McDaniel and Einstein's selection was biased towards smaller age-related declines, as their Table 7.4 omitted most of the studies of ProM proper, omitted over 50% of all studies of ProM, and ignored methodological artifacts such as ceiling effects, intelligence confounds, and ongoing task ease confounds. Similarly, Kliegel et al. 's [25] meta-analysis suffered from a number of shortcomings including the omission of many published studies, and failure to consider methodological artifacts such as ceiling effects, intelligence, confounds, and ongoing task ease confounds (see above for details). Thus, when confounded studies are removed and the data are analyzed separately for ProM proper and for vigilance, the current meta-analysis show large age-related declines in ProM proper with focal cues and smaller but still large age-related declines in vigilance with focal cues.

The present meta-analysis revealed that although the age-related declines in vigilance were numerically smaller with focal vs. non-focal cues, this difference was not statistically significant due to the small size of the difference. In contrast, age-related declines in ProM proper were numerically smaller for the non-focal vs. focal cues, but the statistical comparison would not be meaningful due to the small number of studies that have assessed ProM proper with non-focal cues. Considering the small and inconsistent effects of focal vs. non-focal cues on the size of age-related declines, the focal vs. non-focal cue distinction is unlikely to explain the “perplexing pattern” (i.e., many studies finding age-related declines but some finding no age-related declines) [48]. In addition, it is important to note that smaller age-related declines with focal vs. non-focal cues are consistent with all theories of prospective memory and aging including Craik's [9], [10] account, Maylor's [39] task appropriate processing account, Meier and Graf's [49] transfer appropriate processing account, and McDaniel and Einstein's multiprocess view [50], and thus, do not favor any particular theory. However, only McDaniel and Einstein's multiprocess view [13], [14] predicts no age-related declines with focal cues.

It has been argued, however, that to study age differences in prospective memory properly one ought to make the ongoing task easier for older vs. younger adults. Einstein and McDaniel [11] explained that they made their ongoing task easier for older vs. younger adults because “this [making word lists shorter for older vs. younger adults] equated functional difficulty” of the ongoing task. Similarly, Kvavilashvili et al. [37] explained: “in order to properly assess age effects on prospective memory it is necessary to ensure that both age groups have equal amounts of attentional resources available for the execution of prospective memory task.” Thus, one may argue that the current meta-analysis actually supports McDaniel and Einstein's multiprocess view because the studies that confounded age with the ease of ongoing task and/or verbal intelligence actually resulted in no statistically significant age-related declines (barely missing the conventional alpha  = 0.05).

As discussed by Uttl [2], however, this line of reasoning is specious. First, functionally equating ongoing task difficulty or demands appears difficult, if not impossible, by simply making an the ongoing task easier for older adults in some arbitrary way. To illustrate, Einstein and McDaniel [11] shortened word lists on their working memory task for older vs. younger adults by one item to make them equally difficult but they did not succeed: older adults actually outperformed (significantly) younger adults. Second, ensuring that both age groups have “equal amounts of attentional resources available [left over] for the execution of prospective memory task” is even more daunting without some accurate measure of the left-over resources. Equal performance on the ongoing task does not mean that the amount of left-over resources is the same for younger and older adults and arbitrarily making the ongoing task easier for older vs. younger adults is unlikely to achieve this objective. Third, younger and older adults need not use the same resource pools to achieve the same level of performance, rendering the entire exercise focused on a single resource pool rather superfluous. Fourth, any attempt to equate “functional difficulty” of an ongoing task is likely to have limited ecological validity and real-life relevance. To illustrate, imagine some younger and older adults, all of whom have made plans to buy groceries en route home, traveling by the same bus from work (a university) past the supermarket (ProM cue) to their homes in the city center. Slowing down the ongoing task for older vs. younger adults would be equivalent to slowing down the progress (in time and space) of the seats occupied by older adults relative to the progress of the seats occupied by younger adults along the bus route while keeping all the seats on the same bus. Presently, this seems impossible. Fifth, the current meta-analysis shows that for ProM proper with focal cues, age-related declines are large even though the ongoing task demands in most of these studies were zero as these studies did not include any resource demanding ongoing tasks. For example, participants were listening to an experimenter saying “this is the end of the task” (ProM cue), doing nothing else, having all of their resources available to them, and yet large age-related declines emerged [21].

Accordingly, smaller age-related declines in confounded studies favoring older adults should not be interpreted as showing “no age-related declines with focal cues”. A more appropriate description of these findings is: “If the ongoing task is made much easier for older vs. younger adults and/or if older adults are much smarter than younger adults, then age decline in ProM with focal cues is reduced.” Indeed, these findings parallel those found with retrospective memory. For example, performance on recall tests declines substantially when attentional resources are divided at retrieval (e.g., [51]) and, thus, one can easily eliminate age-related declines in recall by dividing attention for younger adults more than for older adults at retrieval (this confounding would be appropriate because it would “equalize” available resources to younger and older adults for retrieval of previously learned words, following Kvavilashvili et al. 's [37] reasoning and applying it to retrospective memory age-related declines). Similarly, given the moderately strong correlations between recall and verbal intelligence (e.g., [26], [52]), one can easily eliminate age-related declines in recall by comparing less intelligent younger adults with more intelligent older adults.

One could argue that the current study's findings depend on accurate classification of ProM cues as focal vs. non-focal based on McDaniel and Einstein's description of the characteristics of these cues, and that McDaniel and Einstein would classify the cues differently. The analyzes of inter-rater agreement between the cue classification in the current meta-analysis and the classification of cues by McDaniel and Einstein themselves (reported in the method section) set aside these concerns: the inter-rater agreement was very high using both the percentage agreement as well as Krippendorff's alpha measures.

The present meta-analysis has several limitations. First, the high prevalence of ceiling effects in ProM studies has likely reduced the estimated effect sizes even though modeling and *d*_probit_ were used for estimation. Second, the estimated effect sizes are limited by the low reliability of binary indexes of ProM used in primary studies [2], [3]. This low reliability of ProM measurement is also expected to reduce estimated effect sizes. Third, the operational definition of ProM proper vs. vigilance used in this meta-analysis classified age contrasts as ProM proper if there was an intervening task or a delay between ProM instructions and start of an ongoing task. However, it is possible that performance in some studies classified as ProM proper was more dependent on vigilance as these studies used multiple cues. Once a participant responds to one of the cues, he or she may start monitoring for cues and performance may reflect primarily vigilance rather than ProM proper [2], [20]. Fourth, many reports included in the meta-analysis did not provide any assessment of participants' verbal intelligence. Thus, it is possible that intelligence confounds were also present in some of the studies classified as not confounded by verbal intelligence in the present meta-analysis. Finally, to my knowledge, there have been no longitudinal studies of age changes in ProM to date that would verify decline in memory prospectively and all studies to date used cross-sectional design. It is possible, although unlikely, that the pattern of findings could be different in longitudinal studies.

### Conclusions

The current meta-analysis represents a substantial advancement over the previous meta-analyses of age-related declines in ProM with focal vs. non-focal cues. First, the present findings are supported by three meta-analytic approaches – graphical model fitting methods, robust count methods, and the more traditional meta-analysis based on *d*_probit_ effect size indexes. Second, the present meta-analysis is more comprehensive by doubling to tripling the number of included studies relative to the previous meta-analyses by Eintein and McDaniel [14], Uttl [2] (formal meta-analysis of McDaniel and Einstein's meta-analysis of age-declines with focal vs. non-focal cues), and Kliegel et al. [25]. Third, the current meta-analysis did not combine non-confounded with confounded studies, nor ProM proper with vigilance studies, but rather analyzed them separately, which is necessary if one wishes to learn about age-related declines in ProM proper vs. vigilance, rather than age-related declines in a particular blend of ProM proper and vigilance, and non-confounded and confounded studies.

Lastly, this study highlights that age-related declines in ProM with focal cues are large, that even age-related declines in ProM with focal cues can vary across ProM subdomains with large age-related declines in ProM proper and smaller but substantial declines in vigilance, and that age-related declines in ProM proper with focal cues are as large as or even larger than age-related declines in retrospective memory. In turn, these results are consistent with Craik's [9], [10] proposal that age-related declines on ProM tasks are generally large, as large as age-related declines in recall measures of retrospective memory, and vary with the degree of environmental support (i.e., larger on ProM proper vs. vigilance/monitoring). The results support the distinction between ProM proper vs. vigilance/monitoring (see Brandimonte [53], Graf and Uttl [1], Uttl [2], [3]); they highlight the need for authors to explicitly and openly distinguish between ProM proper and vigilance/monitoring, rather than requiring the reader to pore over the method section with a fine-toothed comb to find out whether a particular study investigated vigilance/monitoring or ProM proper (e.g., [6]–[8]). The results directly contradict Einstein, McDaniel, and their colleagues' claims that ProM with focal cues is spared by aging [13], [14], [54]. Finally, the results strongly suggest that the distinction between ProM proper vs. vigilance/monitoring, age confounds, ceiling effects, and low statistical power are responsible for what some have called a “perplexing pattern” (lack of age-related declines in some studies vs. strong age-related declines in other studies) of age-related declines [48]. The “perplexing pattern” is not perplexing at all; it is due to methodological problems and conceptual confusions that have plagued ProM research.

## References

[1] Graf P, Uttl B (2001). Prospective memory: A new focus for research.. Consciousness Cognition.

[2] Uttl B (2008). Transparent meta-analysis of prospective memory and aging.. PLoS ONE.

[3] Uttl B (2005). Age-Related Changes in Event-Cued Prospective Memory Proper.. Ohta N, MacLeod CM, Uttl B, eds.

[4] Harris JE, Haris JE, Morris PE (1984). Remembering to do things: A forgotten topic.. Everyday memory. Actions and absentmindedness.

[5] Uttl B (2005). Measurement of Individual Differences: Lessons from Memory Assessment in Research and Clinical Practice.. Psychological Science.

[6] McDaniel M, Guynn M, Einstein G, Breneiser J (2004). Cue-Focused and Reflexive-Associative Processes in Prospective Memory Retrieval.. Journal of Experimental Psychology.

[7] Marsh R, Hicks J, Hancock T, Munsayac K (2002). Investigating the output monitoring component of event-based prospective memory performance.. Memory & Cognition.

[8] Shapiro S, Krishnan H (1999). Consumer memory for intentions: A prospective memory perspective.. Journal of Experimental Psychology.

[9] Craik FIM (1983). On the Transfer of Information from Temporary to Permanent Memory. Philosophical Transactions of the Royal Society of London.. Series B, Biological Sciences (1934-1990).

[10] Craik FIM, Klix F, Hagendorf H (1986). A functional account of age differences in memory.. Human Memory and Cognitive Capabilities. Mechanisms and Performance.

[11] Einstein G, McDaniel M (1990). Normal aging and prospective memory.. Journal of Experimental Psychology.

[12] McDaniel M, Einstein G, Stout A, Morgan Z (2003). Aging and Maintaining Intentions Over Delays: Do It or Lose It.. Psychology and Aging.

[13] McDaniel M, Einstein G, Rendell P, Matthias Kliegel (2008). The puzzle of inconsistent age-related declines in prospective memory: A multiprocess explanation..

[14] McDaniel M, Einstein G (2007). Prospective memory: An overview and synthesis of an emerging field.

[15] Rendell P, McDaniel M, Forbes R, Einstein G (2007). Age-related effects in prospective memory are modulated by ongoing task complexity and relation to target cue.. Aging, Neuropsychology, and Cognition.

[16] Maylor E, Maria Brandimonte (1996). Does prospective memory decline with age?.

[17] Rendell P, Thomson D (1999). Aging and prospective memory: Differences between naturalistic and laboratory tasks..

[18] Dobbs A, Rule B (1987). Prospective memory and self-reports of memory abilities in older adults.. Journal of Psychology.

[19] Kliegel M, Eschen A, Thone-Otto AI (2004). Planning and realization of complex intentions in traumatic brain injury and normal aging.. Brain and Cognition.

[20] Graf P, Uttl B, Dixon RA, Graf P, Ohta N (2002). Prospective and Retrospective Memory in Adulthood.. Lifespan Development of Human Memory.

[21] Uttl B, Graf P, Miller J, Tuokko H (2001). Pro and retrospective memory in late adulthood.. Conscious Cogn.

[22] Salthouse T, Berish D, Siedlech K (2004). Construct validity and age sensitivity of prospective memory.. Memory & Cognition.

[23] West RL, Gruneberg MM, Morris PE, Sykes RN (1988). Prospective memory and aging.. Practical aspects of memory. Current research and issues, Vol. 1: Memory in everyday life.

[24] Tombaugh T, Grandmaison L, Schmidt J (1995). Prospective memory: Relationship to age and retrospective memory in the Learning and Memory Battery (LAMB).. Clinical Neuropsychologist.

[25] Kliegel M, Jager T, Phillips L (2008). Adult age differences in event-based prospective memory: A meta-analysis on the role of focal versus nonfocal cues.. Psychology and Aging.

[26] Uttl B (2006). Age-related changes in event-cued visual and auditory prospective memory proper.. Aging, Neuropsychology, and Cognition.

[27] Sanchez-Meca J, Marin-Martinez F, Chacon-Moscoso S (2003). Effect-Size Indices for Dichotomized Outcomes in Meta-Analysis.. Psychological Methods.

[28] Birt A (2003). Prospective memory: A distinct form of remembering? Evidence from task comparisons and normal aging. Dissertation Abstracts International. Section B: The Sciences and Engineering..

[29] Henry J, MacLeod M, Phillips L, Crawford J (2004). A Meta-Analytic Review of Prospective Memory and Aging.. Psychology and Aging.

[30] Mantyla T, Nilsson L (1997). Remembering to remember in adulthood: A population-based study on aging and prospective memory.. Aging, Neuropsychology, and Cognition.

[31] Folstein MF, Folstein SE, McHugh PR (1975). “Mini-mental state”. A practical method for grading the cognitive state of patients for the clinician.. J Psychiatr Res.

[32] Martin M, Kliegel M, McDaniel M (2003). The involvement of executive functions in prospective memory performance of adults.. Journal of Psychology.

[33] Bailey P, Henry J, Rendell P, Phillips L, Kliegel M (2010). Dismantling the “age-prospective memory paradox”: The classic laboratory paradigm simulated in a naturalistic setting.. Journal of Experimental Psychology.

[34] Logie R, Maylor E (2009). An Internet study of prospective memory across adulthood.. Psychology and Aging.

[35] Cherry K, LeCompte D (1999). Age and individual differences influence prospective memory.. Psychology and Aging.

[36] Cherry K, Martin R, Simmons-D'Gerolamo S, Pinkston J, Griffing A (2001). Prospective remembering in younger and older adults: Role of the prospective cue.. Memory.

[37] Kvavilashvili L, Kornbrot D, Mash V, Cockburn J, Milne A (2009). Differential effects of age on prospective and retrospective memory tasks in young, young-old, and old-old adults.. Memory.

[38] Cherry K, Plauche M, Serge P (2003). Age differences in prospective memory: Role of task complexity and prospective support.. Shohov.

[39] Maylor E (1996). Age-related impairment in an event-based prospective-memory task.. Psychology and Aging.

[40] Reese C, Cherry K (2002). The effects of age, ability, and memory monitoring on prospective memory task performance.. Aging, Neuropsychology, and Cognition.

[41] Farrimond S, Knight RG, Titov N (2006). The effects of aging on remembering intentions: performance on a simulated shopping task.. Applied Cognitive Psychology.

[42] Krippendorff K (2004). Content analysis: an introduction to its methodology..

[43] Davidson A, Hinkley D (1997). Bootstrap Methods and Their Application..

[44] Higgins JPT, Thompson SG, Deeks JJ, Altman DG (2003). Measuring inconsistency in meta-analyses.. BMJ.

[45] Spencer WD, Raz N (1995). Differential effects of aging on memory for content and context: A meta-analysis.. Psychology and Aging.

[46] La Voie D, Light LL (1994). Adult age differences in repetition priming: A meta-analysis.. Psychology and Aging.

[47] Verhaeghen P, Marcoen A, Goossens L (1993). Facts and fiction about memory aging: a quantitative integration of research findings.. J Gerontol.

[48] Einstein G, McDaniel M (2005). Prospective memory: Multiple retrieval processes.. Current Directions in Psychological Science.

[49] Meier B, Graf P (2000). Transfer appropriate processing for prospective memory tests.. Applied Cognitive Psychology.

[50] McDaniels M, Einstein G (2000). Strategic and automatic processes in prospective memory retrieval: A multiprocess framework.. http://dx.doi.org/10.1002/acp.775.

[51] Fernandes MA, Moscovitch M (2000). Divided attention and memory: Evidence of substantial interference effects at retrieval and encoding.. Journal of Experimental Psychology: General.

[52] Uttl B, Graf P, Richter LK (2002). Verbal Paired Associates tests limits on validity and reliability.. Arch Clin Neuropsychol.

[53] Brandimonte M, Ferrante D, Feresin C, Delbello R (2001). Dissociating prospective memory from vigilance processes.. Psicologica.

[54] Einstein G, McDaniel M, Thomas R, Mayfield S, Shank H (2005). Multiple Processes in Prospective Memory Retrieval: Factors Determining Monitoring Versus Spontaneous Retrieval.. Journal of Experimental Psychology.

[55] Huppert F, Johnson T, Nickson J (2000). High prevalence of prospective memory impairment in the elderly and in early-stage dementia: Findings from a population-based study.. http://dx.doi.org/10.1002/acp.771.

[56] Cockburn J, Smith P (1994). Anxiety and errors of prospective memory among elderly people.. Journal of Psychology.

[57] Kliegel M, McDaniel M, Einstein G (2000). Plan formation, retention, and execution in prospective memory: A new approach and age-related effects.. Memory & Cognition.

[58] Cuttler C, Graf P (2007). Personality predicts prospective memory task performance: An adult lifespan study.. Journal of Psychology.

[59] Duchek J, Balota D, Cortese M (2006). Prospective memory and apolipoprotein e in healthy aging and early stage Alzheimer's disease.. Neuropsychology.

[60] Kliegel M, Mackinlay R, Jager T (2008). Complex prospective memory: Development across the lifespan and the role of task interruption.. Developmental Psychology.

[61] Skladzien E (2010). Age differences in output-monitoring accuracy.. Aging, Neuropsychology, and Cognition.

[62] Zimmermann T, Meier B (2006). The rise and decline of prospective memory performance across the lifespan.. Journal of Experimental Psychology.

[63] Cohen A, West R, Craik FI (2001). Modulation of the prospective and retrospective components of memory for intentions in younger and older adults.. Aging, Neuropsychology, and Cognition.

[64] d'Ydewalle G, Luwel K, Brunfaut E (1999). The importance of on-going concurrent activities as a function of age in time- and event-based prospective memory.. Journal of Cognitive Psychology.

[65] Einstein G, McDaniel M, Richardson S, Guynn M, Cunfer A (1995). Aging and prospective memory: Examining the influences of self-initiated retrieval processes.. Journal of Experimental Psychology.

[66] Einstein G, Smith R, McDaniel M, Shaw P (1997). Aging and prospective memory: The influence of increased task demands at encoding and retrieval.. Psychology and Aging.

[67] Einstein G, McDaniel M, Smith R, Shaw P (1998). Habitual prospective memory and aging: Remembering instructions and forgetting actions.. Psychological Science.

[68] Logie R, Maylor E, Della Sala S, Smith G (2004). Working memory in event- and time-based prospective memory tasks: Effects of secondary demand and age.. Journal of Cognitive Psychology.

[69] Maylor E, Smith G, Della Sala S, Logie R (2002). Prospective and retrospective memory in normal aging and dementia: An experimental study.. Memory & Cognition.

[70] Vogels WW, Dekker M, Brouwer W, de Jong R (2002). Age-related changes in event-related prospective memory performance: A comparison of four prospective memory tasks.. Brain and Cognition.

[71] West R, Craik FI (2001). Influences on the efficiency of prospective memory in younger and older adults.. Psychology and Aging.

[72] d'Ydewalle G, Bouckaert D, Brunfaut E (2001). Age-related differences and complexity of ongoing activities in time- and event-based prospective memory.. Journal of Psychology.

[73] Kidder D, Park D, Hertzog C, Morrell R (1997). Prospective memory and aging: The effects of working memory and prospective memory task load.. Aging, Neuropsychology, and Cognition.

[74] Kliegel M, Jager T (2006). Delayed-Execute Prospective Memory Performance: The Effects of Age and Working Memory.. Developmental Neuropsychology.

[75] Mantyla T (1993). Priming effects in prospective memory.. Memory.

[76] Mantyla T (1994). Remembering to remember: Adult age differences in prospective memory.. Journals of Gerontology.

[77] Maylor E (1993). Aging and forgetting in prospective and retrospective memory tasks.. Psychology and Aging.

[78] Maylor E (1998). Changes in event-based prospective memory across adulthood.. Aging, Neuropsychology, and Cognition.

[79] Park D, Hertzog C, Kidder D, Morrell R, Mayhorn C (1997). Effect of age on event-based and time-based prospective memory.. Psychology and Aging.

[80] West R, Covell E (2001). Effects of aging on event-related neural activity related to prospective memory.. NeuroReport: For Rapid Communication of Neuroscience Research.

[81] West R, Herndon R, Covell E (2003). Neural correlates of age-related declines in the formation and realization of delayed intentions.. Psychology and Aging.

[82] West R, Bowry R (2005). Effects of aging and working memory demands on prospective memory.. Psychophysiology.

[83] West R, Craik FI (1999). Age-related decline in prospective memory: The roles of cue accessibility and cue sensitivity.. Psychology and Aging.

[84] Zollig J, West R, Martin M, Altgassen M, Lemke U (2007). Neural correlates of prospective memory across the lifespan.. Neuropsychologia.

[85] Cohen A, Dixon R, Lindsay D, Masson ME (2003). The Effect of Perceptual Distinctiveness on the Prospective and Retrospective Components of Prospective Memory in Young and Old Adults.. Journal of Experimental Psychology.

[86] Einstein G, Holland L, McDaniel M, Guynn M (1992). Age-related deficits in prospective memory: The influence of task complexity.. Psychology and Aging.

[87] Jager T, Kliegel M (2008). Time-based and event-based prospective memory across adulthood: Underlying mechanisms and differential costs on the ongoing task.. Journal of General Psychology.

[88] McDermott K, Knight R (2004). The Effects of Aging on a Measure of Prospective Remembering using Naturalistic Stimuli.. Applied Cognitive Psychology.

[89] Bastin C, Meulemans T (2002). Are time-based and event-based prospective memory affected by normal aging in the same way?.

[90] Knight R, Nicholls J, Titov N (2008). The effects of old age and distraction on the assessment of prospective memory in a simulated naturalistic environment.. International Psychogeriatrics.

[91] Marsh R, Hicks J, Cook G, Mayhorn C (2007). Comparing older and younger adults in an event-based prospective memory paradigm containing an output monitoring component.. Aging, Neuropsychology, and Cognition.

